# Patterns in Physician Burnout in a Stable-Linked Cohort

**DOI:** 10.1001/jamanetworkopen.2023.36745

**Published:** 2023-10-06

**Authors:** Marcus V. Ortega, Michael K. Hidrue, Sara R. Lehrhoff, Dan B. Ellis, Rachel C. Sisodia, William T. Curry, Marcela G. del Carmen, Jason H. Wasfy

**Affiliations:** 1 Division of Female Pelvic Medicine and Reconstructive Surgery, Department of Obstetrics and Gynecology, Massachusetts General Hospital, Harvard Medical School, Boston; 2 Massachusetts General Physicians Organization, Boston; 3 Department of Anesthesiology, Massachusetts General Hospital, Harvard Medical School, Boston; 4 Division of Gynecology Oncology, Department of Obstetrics and Gynecology, Massachusetts General Hospital, Harvard Medical School, Boston; 5 Department of Neurosurgery, Massachusetts General Hospital, Harvard Medical School, Boston; 6 Cardiology Division, Department of Medicine, Massachusetts General Hospital, Harvard Medical School, Boston

## Abstract

**Importance:**

Physician burnout is widely reported to be an increasing problem in the US. Although prior analyses suggest physician burnout is rising nationally, these analyses have substantial limitations, including different physicians joining and leaving clinical practice.

**Objective:**

To examine the prevalence of burnout among physicians in a large multispecialty group over a 5-year period.

**Design, Setting, and Participants:**

This survey study was conducted in 2017, 2019, and 2021 and involved physician faculty members of the Massachusetts General Physicians Organization. Participants represented different clinical specialties and a full range of career stages. The online survey instrument had 4 domains: physician career and compensation satisfaction, physician well-being, administrative workload on physicians, and leadership and diversity.

**Exposure:**

Time.

**Main Outcomes and Measures:**

Physician burnout, which was assessed with the Maslach Burnout Inventory. A binary burnout measure was used, which defined burnout as a high score in 2 of the 3 burnout subscales: Exhaustion, Cynicism, and Reduced Personal Efficacy.

**Results:**

A total of 1373 physicians (72.9% of the original 2017 cohort) participated in all 3 surveys. The cohort included 690 (50.3%) male, 921 (67.1%) White, and 1189 (86.6%) non-Hispanic individuals. The response rates were 93.0% in 2017, 93.0% in 2019, and 92.0% in 2021. Concerning years of experience, the cohort was relatively well distributed, with the highest number and proportion of physicians (478 [34.8%]) reporting between 11 and 20 years of experience. Within this group, burnout declined from 44.4% (610 physicians) in 2017 to 41.9% (575) in 2019 (*P* = .18) before increasing to 50.4% (692) in 2021 (*P* < .001).

**Conclusions and Relevance:**

Findings of this survey study suggest that the physician burnout rate in the US is increasing. This pattern represents a potential threat to the ability of the US health care system to care for patients and needs urgent solutions.

## Key Points

**Question:**

What is the burnout rate among US physicians over the past 5 years?

**Findings:**

In this survey study involving 1373 physicians and 3 survey periods, significantly higher burnout rates were found among female physicians compared with their male counterparts, primary care physicians compared with physicians in other specialties, and physicians with 10 years of experience or less compared with those with more experience.

**Meaning:**

Findings of this study suggest that this burnout pattern is a potential threat to the ability of the US health care system to care for patients and thus needs immediate solutions.

## Teaser

This survey study assesses the prevalence of burnout among active clinical staff members of the Massachusetts General Physicians Organization who participated in a survey in 2017, 2019, and 2021.

## Introduction

Physician burnout is described as a state of emotional, physical, and mental exhaustion caused by prolonged stress in the workplace.^1^ The causes of burnout are multifactorial,^2^ including loss of autonomy,^3^ high workload,^4^ and poor work-life balance.^5^ Physicians with burnout are more likely to make medical errors,^6^ have lower patient satisfaction scores,^7,8,9^ and have higher rates of absenteeism.^10^ Burnout exacerbates the already existing shortage of physicians in certain areas and specialties, leading to longer wait times and decreased access to care for patients.^11^ Furthermore, burnout is also associated with a reduction in the overall quality of care.^12^

Available evidence suggests that the physician burnout rate is increasing. A large national survey study from nearly 10 years ago found an alarming level of physician burnout, with 45.8% of physicians reporting at least 1 symptom of burnout when assessed with the Maslach Burnout Inventory (MBI).^5^ During the COVID-19 pandemic, a few studies described a sharp increase in burnout rates in the US. Shanafelt et al^13^ found that toward the end of the second year of the pandemic, surveyed physicians reported significantly higher mean emotional exhaustion and depersonalization scores compared with scores observed in 2011, 2014, 2017, and 2020 (all *P* < .001).

Although the physician burnout rate appears to be increasing nationally, analyses of burnout are often subject to multiple limitations that are common to survey methods. First, physicians with more years of experience had less burnout; thus, cross-sectional surveys suggest that increased burnout may be at least partially due to physicians of a certain age retiring.^14^ Second, survey data only reflect data from those who answered the survey. Specifically, the largest series survey response rates in prior analyses ranged from 26.7% to 32.0%.^5,6^ Physicians who did not respond to the survey may be feeling more or less burned out compared with those who responded, and these proportions may change over time. Third, if physicians with a high level of burnout are more likely to leave medicine, the burnout pattern among those who stay may be worse than previously reported. As such, it is unclear whether reports of increasing physician burnout are biased. The purpose of this survey study was to examine the prevalence of burnout among physicians in a large multispecialty group over a 5-year period.

## Methods

We surveyed physician faculty members of the Massachusetts General Physicians Organization (MGPO) to explore burnout rates over time across different specialties. The MGPO Institutional Review Board approved this study. Participating physicians provided written consent by filling out the survey. We followed the American Association for Public Opinion Research (AAPOR) reporting guideline.

The present survey was conducted as part of the MGPO’s biannual survey to gauge physician perceptions of the functioning of the clinical enterprise within and across departments, measure progress on organizational priorities, and evaluate hospital leadership. All active clinical staff members of the MGPO were invited to participate in the survey. Participants were compensated for their effort in the amount of $850. Specialties in internal medicine included nephrology, pulmonary and critical care, rheumatology, allergy immunology, cardiology, endocrinology, gastroenterology, hematology oncology, infectious disease, palliative care, neurology, pediatric specialists, physical medicine and rehabilitation, physiatry, and radiation oncology. While primary care physicians (PCPs) often are under internal medicine, their day-to-day responsibilities, patient interactions, and challenges can differ significantly from those of other specialists within the internal medicine department. By analyzing PCPs as a separate group, we aimed to identify the particular aspects of PCP work that may be associated with increased or decreased burnout.

In general, the MGPO survey uses a large financial incentive to maintain a response rate of greater than 90%. A response rate higher than 90% minimizes the potential impact of missing data. Although the limited data set is deidentified, physicians can be linked over time before deidentification so that those entering or exiting the data set can be excluded. Furthermore, the study population includes a full range of career stages and clinical specialties in the largest multispecialty group in New England and one of the largest multispecialty groups in the US. To assess reports of increasing physician burnout over time, we examined the prevalence of burnout among different physician groups in 2017, 2019, and 2021.

### Survey Instruments and Variables

The online survey instrument adhered to the AAPOR guidelines for reporting survey studies.^15^ It covered personal and professional characteristics (eg, sex, race and ethnicity, years of experience [measured as number of years since training], specialty, and trusted advisor), well-being metrics (eg, overall career satisfaction, burnout, tolerance of uncertainty, work engagement, professional fulfillment, and peer support), financial compensation, administrative workload, leadership, and diversity.^14,16^ Race and ethnicity were self-reported by participants and included the following categories: American Indian and Alaska Native, Asian, Black, Hispanic, non-Hispanic, White, others, and not reported. Race and ethnicity data were collected and analyzed because we would like to evaluate the physician burnout rates among different racial groups.

The survey encompassed 4 domains: (1) physician career and compensation satisfaction; (2) physician well-being, assessed using the MBI and Utrecht Work Engagement Scale; (3) administrative workload on physicians; and (4) leadership and diversity content.^1^ Overall, the MBI evaluates physician burnout using 3 subscales: Exhaustion, Cynicism, and Professional Efficacy.^1^ The MBI has been extensively researched and shown to have good reliability and validity, making it a well-accepted tool for assessing burnout. It includes 22 items, and respondents are asked to rate each item on a 7-point frequency scale that ranges from 0 (never) to 6 (every day). As described in the MBI manual, a score of 3.2 or higher on the Exhaustion Subscale, 2.6 or higher on the Cynicism Subscale, or 3.8 or lower on the Professional Efficacy Subscale denotes a high level of burnout for the respondent in that subscale.^17^

For this analysis, we used a binary burnout measure, which defined burnout as a high score in 2 of the 3 burnout subscales: Exhaustion, Cynicism, and Reduced Personal Efficacy. While burnout was examined as the binary outcome, the underlying measure was continuous. To address ways by which the variable specification might affect the results, we specified, in a sensitivity analysis, a separate model for each of the burnout subscales (the continuous outcomes).

### Statistical Analysis

We used a 2-stage hierarchical regression model with a physician as a random variable to account for the longitudinal nature of the data. For the binary outcome, we specified a hierarchical logistic model. For the continuous outcomes (burnout subscales), we specified a generalized linear model with a log link and γ distribution. This framework allowed us to control for the association of outcomes from the same physician over the study period and to estimate efficient parameters of the risk factors in the model. It also allowed us to decompose the residual variance in burnout into variation between physicians and variation within physicians over the study period. The residual variance between physicians represents the role of unobserved physician characteristics in burnout. These physician characteristics include, for example, the differences in personal resilience. Similarly, the residual variance within physicians represents the role of unobserved factors that change over the study period. These overall factors include work environment–related factors that are associated with burnout. We used intraclass correlation to decompose total variation into the 2 components for the continuous models and median odds ratio (OR) for the binary model.^18^ All models adjusted for the same set of risk factors: sex, years of experience, specialty, and survey year. Survey year, with 2019 as the reference, was a measure of how much the COVID-19 pandemic might have been associated with physician burnout.

We used standard descriptive statistics to summarize the data by demographic and professional variables. All statistical tests were 2-sided, and *P* < .05 were considered to be statistically significant. Regression results are reported as ORs or rate ratios (RRs), depending on whether the outcome is binary or continuous. All statistical analyses were performed using SAS, version 9.4 (SAS Institute Inc).

## Results

A total of 1373 physicians (72.9% of the original 2017 cohort) participated in all 3 surveys. These physicians included 690 males (50.3%) and 579 females (42.2%), most of whom identified as White (921 [67.1%]) and non-Hispanic (1189 [86.6%]) individuals (Table 1). Concerning years of experience, the cohort was relatively well distributed, with the largest proportion of respondents (478 [34.8%]) reporting between 11 and 20 years of experience. The response rates were 93.0% in 2017, 93.0% in 2019, and 92.0% in 2021. Within this group, the burnout rate decreased from 44.4% (610 respondents) in 2017 to 41.9% (575) in 2019 (*P* = .18) and then increased to 50.4% (692) in 2021 (*P* < .001). This pattern was consistent across all demographic variables and specialty groups (Table 1).

**Table 1.** Distribution of Physicians by Demographic Characteristics

Covariate	All physicians, No. (%)	Burnout rate by survey year, No. (%)		
		2017	2019	2021
Sex				
Female	579 (42.2)	269 (46.5)	261 (45.1)	331 (57.2)
Male	690 (50.3)	289 (41.9)	261 (37.8)	296 (42.9)
Preferred not to say	104 (7.6)	52 (50.0)	53 (51.0)	65 (62.5)
Race^a^				
American Indian and Alaska Native	6 (0.4)	5 (83.3)	4 (66.8)	4 (66.7)
Asian	213 (15.5)	96 (45.1)	104 (48.8)	115 (54.0)
Black	25 (1.8)	15 (60.0)	9 (36.0)	13 (52.0)
White	921 (67.1)	396 (43.0)	359 (39.0)	448 (48.6)
Other^b^	64 (4.7)	27 (42.2)	25 (39.1)	28 (43.8)
Preferred not to say	144 (10.5)	71 (49.3)	74 (51.4)	84 (58.3)
Ethnicity^a^				
Hispanic	60 (4.4)	26 (43.3)	23 (38.3)	29 (48.3)
Non-Hispanic	1189 (86.6)	527 (44.3)	488 (41.0)	587 (49.3)
Preferred not to say	124 (9.0)	57 (46.0)	64 (51.6)	76 (61.3)
Years of experience				
≤10	360 (26.2)	269 (46.7)	232 (48.7)	204 (56.7)
11-20	478 (34.8)	185 (45.0)	204 (44.8)	252 (52.7)
21-30	324 (23.6)	112 (44.1)	105 (37.1)	162 (50.0)
>30	211 (15.4)	44 (33.3)	34 (21.4)	74 (35.1)
Specialty				
ERAP	248 (18.1)	101 (40.1)	107 (43.0)	118 (47.6)
Internal medicine	746 (54.3)	314 (42.3)	303 (40.7)	360 (48.3)
Primary care	218 (15.9)	126 (57.5)	104 (47.7)	140 (64.2)
Surgery	161 (11.7)	69 (43.1)	61 (37.9)	74 (46.0)
Burnout subscales				
High Exhaustion	NA	783 (57.0)	680 (49.5)	821 (59.8)
High Cynicism	NA	679 (49.5)	654 (47.6)	771 (56.2)
Low Personal Efficacy	NA	266 (19.4)	295 (21.5)	338 (24.6)
Overall burnout	NA	610 (44.4)	575 (41.9)	692 (50.4)

Abbreviations: ERAP, emergency medicine, radiology, anesthesia, and pathology; NA, not applicable.

^a^ Race and ethnicity were self-reported by participants in the survey.

^b^ Other category included Native Hawaiian or Other Pacific Islander.

Most physicians stayed in the same state of burnout throughout the 3 periods. We found that 26.7% of physicians experienced burnout, scoring high in 2 of the 3 burnout subscales in all 3 surveys, whereas 35.4% of physicians did not reach high burnout scores in any of the surveys. When we examined discrete elements of the burnout scale, we found that over 30% of physicians increased their high score status, whereas over 60% of physicians did not change their score during the study period (Figure).

**Figure.**
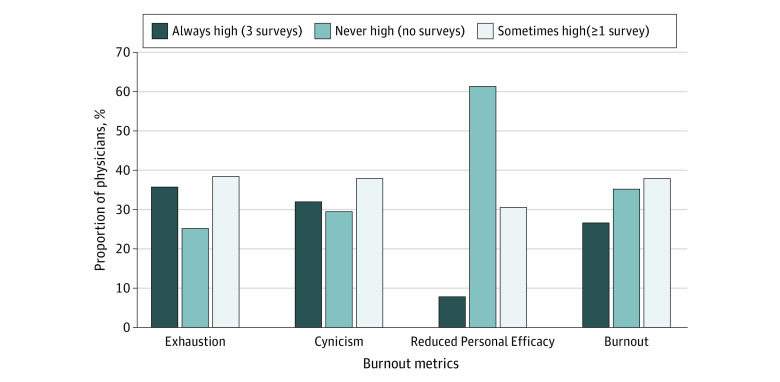
Distribution of Respondents by Frequency of High Score Across 3 Survey Periods

Table 2 presents the regression results using the binary burnout measure. The burnout rate for 2017 was not statistically different from the 2019 rate (OR, 1.15; 95% CI, 0.94-1.41). However, compared with 2019, the burnout rate increased significantly in 2021 (OR, 2.10; 95% CI, 1.70-2.60). Among other risk factors, sex, specialty, and years of experience were significantly associated with burnout. Female physicians had a higher burnout rate than male physicians (OR, 1.47; 95% CI, 1.02-2.12). Primary care physicians had a higher burnout rate than physicians in internal medicine (OR, 2.82; 95% CI, 1.76-4.50). Physicians with more experience had a lower burnout rate than those with 10 years of experience or less (eg, for physicians with >30 years of experience: OR, 0.21; 95% CI, 0.13-0.35) (Table 2). There was no meaningful difference in parameter estimates between the simple and hierarchical logistic models. The median OR for the hierarchical logistic model was 8.90 (95% CI, 7.20-10.80), consistent with the significant variation between individual physicians.

**Table 2.** Odds Ratio Estimates of Burnout From 2017 to 2021

Covariate	OR (95% CI)	
	Hierarchical logistic model	Simple logistic model
Sex		
Female	1.47 (1.02-2.12)	1.20 (1.05-1.37)
Male	1 [Reference]	1 [Reference]
Preferred not to say	1.78 (0.67-4.76)	1.36 (0.94-1.97)
Race		
Asian	1.29 (0.79-2.10)	1.13 (0.95-1.36)
Black	1.15 (0.36-1.93)	1.10 (0.69-1.76)
White	1 [Reference]	1 [Reference]
Others	0.91 (0.43-1.93)	0.94 (0.71-1.26)
Preferred not to say	1.45 (0.61-3.46)	1.21 (0.88-1.66)
Years of experience		
≤10	1 [Reference]	1 [Reference]
11-20	0.73 (0.53-1.01)	0.86 (0.74-1.00)
21-30	0.55 (0.37-0.83)	0.73 (0.61-0.87)
≥30	0.21 (0.13-0.35)	0.40 (0.32-0.51)
Specialty		
ERAP	0.96 (0.61-1.53)	0.97 (0.82-1.16)
Internal medicine	1 [Reference]	1 [Reference]
Primary care	2.82 (1.76-4.50)	1.80 (1.50-2.16)
Surgery	0.93 (0.54-1.60)	0.96 (0.78-1.18)
Survey year		
2017	1.15 (0.94-1.41)	1.08 (0.93-1.26)
2019	1 [Reference]	1 [Reference]
2021	2.10 (1.70-2.60)	1.50 (1.28-1.74)
Median OR (95% CI)	8.90 (7.20-10.80)	NA

Abbreviations: ERAP, emergency medicine, radiology, anesthesia, and pathology; NA, not applicable; OR, odds ratio.

Table 3 presents regression results of the continuous measure of burnout subscales. The results generally confirmed the findings from the binary measure of burnout. Compared with 2019, in 2021 the Exhaustion score increased by 20.0% (RR, 1.20; 95% CI, 1.17-1.23), Cynicism score increased by 20.0% (RR, 1.20; 95% CI, 1.16-1.25), and Reduced Personal Efficacy score increased by 9.0% (RR, 1.09; 95% CI, 1.05-1.14). The Exhaustion score was greater by 11.0% (RR, 1.11; 95% CI, 1.05-1.18) among female physicians compared with their male peers. Primary care physicians had a 25.0% (RR, 1.25; 95% CI, 1.17-1.34) higher Exhaustion score and 20.0% (RR, 1.20; 95% CI, 1.09-1.31) higher Cynicism score than physicians in internal medicine (Table 3). The intraclass correlation showed that 61.0% of the total variation in Exhaustion, 53.0% of the total variation in Cynicism, and 51.0% of the total variation in Reduced Personal Efficacy were variations between as opposed to within physicians in different periods.

**Table 3.** Rate Ratio Estimates of Burnout Subscales

Covariate	Subscale, RR (95% CI)		
	Exhaustion	Cynicism	Reduced Personal Efficacy
Sex			
Female	1.11 (1.05-1.18)	1.04 (0.96-1.12)	1.07 (0.99-1.15)
Male	1 [Reference]	1 [Reference]	1 [Reference]
Preferred not to say	1.15 (1.03-1.29)	1.23 (1.10-1.38)	1.13 (0.98-1.30)
Years of experience			
≤10	1 [Reference]	1 [Reference]	1 [Reference]
11-20	0.96 (0.92-1.01)	0.99 (0.93-1.06)	0.93 (0.87-0.99)
21-30	0.92 (0.87-0.98)	0.91 (0.84-0.98)	0.83 (0.77-0.90)
>30	0.80 (0.74-0.87)	0.78 (0.71-0.87)	0.72 (0.65-0.80)
Specialty			
ERAP	0.97 (0.89-1.05)	0.97 (0.87-1.07)	1.06 (0.96-1.17)
Internal medicine	1 [Reference]	1 [Reference]	1 [Reference]
Primary care	1.25 (1.17-1.34)	1.20 (1.09-1.31)	1.07 (0.97-1.17)
Surgery	0.97 (0.88-1.07)	0.90 (0.80-1.02)	0.87 (0.78-0.97)
Survey year			
2017	1.13 (1.11-1.16)	1.02 (0.99-1.06)	0.95 (0.92-0.99)
2019	1 [Reference]	1 [Reference]	1 [Reference]
2021	1.20 (1.17-1.23)	1.20 (1.16-1.25)	1.09 (1.05-1.14)
Intraclass correlation	61.0 (NA)	53.0 (NA)	51.0 (NA)

Abbreviations: ERAP, emergency medicine, radiology, anesthesia, and pathology; NA, not applicable; RR, rate ratio.

Burnout rates were compared for each survey year, with the corresponding data presented in Table 4. The analysis revealed no significant differences in burnout rates between physicians who were included in and physicians who were excluded from the sample throughout the observation period. However, a pattern was observed in 2017 and 2019: the excluded group demonstrated marginally higher burnout rates than the included group (2017: 48.9% [250 physicians] vs 44.4% [610], *P* = .08; 2019: 43.9% [281] vs 41.9% [575], *P* = .39) (Table 4).

**Table 4.** Comparing Burnout Rate Among Included and Excluded Physicians^a^

Survey year	Included in study		Excluded from study		*P* value
	No.	Burnout rate, No. (%)	No.	Burnout rate, No. (%)	
2017 (n = 1884)	1373	610 (44.4)	511	250 (48.9)	.08
2019 (n = 2013)	1373	575 (41.9)	640	281 (43.9)	.39
2021 (n = 2078)	1373	692 (50.4)	706	358 (50.7)	.89

^a^ For each survey year, physicians with missing data for at least 1 year were excluded from the comparison.

In another analysis, we examined several baseline characteristics of the sample for potential systematic differences between 2 groups: physicians who did not experience a high level of burnout across all 3 survey years and physicians who experienced a high level of burnout at least once during the study. Those without a high burnout level vs those with a high burnout level tended to have more years of experience since training (17.5 vs 14 years; *P* < .001), spend less time on administrative duties (24.0% vs 31.0% of time in an average week; *P* < .001), be more satisfied with their compensation (47.0% [285] vs 26.2% [201]; *P* < .001), and be female (40.7% [284] vs 31.5% [176]; *P* < .001) and were less likely to be PCPs from primary care (22.4% [49]) rather than from other specialties (37.9% [438]) (*P* < .001). These findings aligned with our understanding of the factors associated with burnout, except for the proportion of female physicians.

## Discussion

In this survey study, we extended prior results that suggested that physician burnout increased significantly during the COVID-19 pandemic.^19^ Controlling for demographic characteristics and specialty risk factors, the Exhaustion and Cynicism scores increased by 20.0% each and Reduced Personal Efficacy increased by 9.0% from 2019 levels. Similarly, the odds of burnout doubled from 2019 to 2021. Some groups had higher burnout rates than others. Female physicians, PCPs, and those with 10 years of experience or less reported more burnout than their peers. These results are important because the data set and study design addressed some of the shortcomings of previous studies. In particular, the surveys in all periods had near-complete response rates, and we used longitudinal data to account for potential ecological bias.

This study highlighted the increased burnout among physicians in the US. There was a large variability in reported prevalence rates of physician burnout, likely due to a lack of agreed terminology and definition. A study by Rotenstein et al^20^ examined the prevalence of burnout reported in 182 studies and involving more than 100 000 individuals and concluded a marked variation in burnout definitions, assessment methods, and study quality. Prior studies reported findings with low response rates that resulted in nonrepresentative samples of physicians. In one of the most extensive studies on physician burnout, of the 27 276 physicians who received an invitation to participate, only 7288 (26.7%) completed the surveys.^21^ The present cohort had high response rates (>90% in the 3 survey years), which can mitigate the risk of selection bias. Furthermore, selection could have skewed the results over time, as previous research indicated that physicians with more years of experience had less burnout than their younger counterparts, and retirements or career changes could have altered the composition of the sample.^14^ The present study followed the same physician group over time to observe the patterns more accurately. As such, the results provide more precise estimates of burnout prevalence among physicians.

The study demonstrated that the burnout rate more than doubled between 2019 and 2021. This finding agrees with results of an extensive pre–COVID-19 survey study of more than 35 000 physicians that showed the worsening of burnout rates, with 54.4% of physicians (3680) reporting at least 1 symptom of burnout in 2014 compared with 45.5% (3310) in 2011 (*P* < .001).^22^ The study by Shanafelt et al^22^ was limited by a relatively low response rate (nearly 20% of surveyed physicians did not respond) and a greater proportion of older vs younger physicians as well as female vs male physicians in the 2014 cohort.

In the US, the COVID-19 pandemic has had profound implications for the physician workforce nationwide.^22^ We found increasing burnout during the pandemic. Pandemic-related uncertainties have also taken an emotional and physical toll at the personal level and affected the labor force scarcity. A study that investigated the workforce impact of the pandemic found that 3 in 4 physicians felt overworked and that half of the physicians were considering a change in employment during the pandemic.^23^

Additionally, the analysis showed that a significant portion of the burnout variance was associated with individual differences among physicians. For example, 35.8% of surveyed physicians had high Exhaustion scores in all 3 survey periods, and 25.4% of physicians never reached high Exhaustion scores. In other words, 61.2% of the sample had the same Exhaustion status throughout the 3 surveys. The scores for Cynicism and overall burnout were similar (Figure). This finding remained the same after we adjusted the model for potential risk factors, including sex, years of experience, specialty, and survey year.

In the sensitivity analysis, we compared burnout rates between physicians with complete data and physicians with missing data for at least 1 survey year. We found no significant differences in burnout rates between the groups (Table 4).

Overall, this study revealed an association between physician burnout and several personal and professional factors. Those with less burnout often had more years of experience, suggesting that familiarity with the profession is associated with reduced stress. These physicians also spent less time on administrative tasks, pointing to increased job satisfaction with less bureaucracy. Greater satisfaction with compensation among physicians with less burnout implied that financial contentment is associated with a lower Exhaustion score. These individuals were also less likely to be PCPs.

### Limitations

Limitations of this study include, first, the limitations inherent to a survey-based design such as the inability to draw causal inferences given that results relied on respondents’ self-reporting. Second, the 2017 survey was conducted a few months after changes to the new electronic medical record system were implemented at Massachusetts General Hospital, and the 2021 survey was conducted in the middle of the COVID-19 pandemic. Third, the findings may not be generalizable at large because the study was conducted in a single academic medical center in Northeastern US. Fourth, although all participating physicians were involved in delivering clinical care, many of them were also engaged in nonclinical activities, such as research, education, and administrative roles, which can affect burnout rates. However, using this unique data source (MGPO survey) allowed us to overcome the limitations of prior studies, including ecological inference bias, changes in the physician workforce, and bias associated with missing data and differential responsiveness. Fifth, the $850 financial incentive may bias the survey results, possibly leading to underreporting of burnout and an overly favorable view of physician well-being within the organization. This factor could hide the true severity of physician burnout, making the burnout situation seem better than it is.

## Conclusions

The findings of this survey study suggest that the physician burnout rate in the US is increasing. This pattern represents a potential threat to the ability of the health care system to care for patients in the US and needs urgent solutions.

## Notes

Supplement.
Data Sharing Statement
