# Supplementary material for: Patterns in Physician Burnout in a Stable-Linked Cohort
Source: JAMA Netw Open. 2023 Oct 6;6(10):e2336745. doi: 10.1001/jamanetworkopen.2023.36745 (PMC10559175; doi:10.1001/jamanetworkopen.2023.36745)
Supplement: Supplement. — Data Sharing Statement [file jamanetwopen-e2336745-s001.pdf]

Data Sharing Statement

Ortega. Patterns in Physician Burnout in a Stable-Linked Cohort. *JAMA Netw Open*. Published October 06, 2023. doi:10.1001/jamanetworkopen.2023.36745

Data
Data available: No
